# Antibiotic prescribing patterns at children’s outpatient departments of primary care institutions in Southwest China

**DOI:** 10.1186/s12875-022-01875-9

**Published:** 2022-10-26

**Authors:** Wenju Wang, Shitao Yu, Xunrong Zhou, Lei Wang, Xun He, Hanni Zhou, Yue Chang

**Affiliations:** 1 School of Public Health, Guizhou Medical University, Guiyang, Guizhou Province China; 2 Guiyang Public Health Clinical Center, Guiyang, Guizhou Province China; 3 Second Affiliated Hospital of Guizhou University of Traditional Chinese Medicine, Guiyang, Guizhou Province China; 4 Primary Health Department of Guizhou Provincial Health Commission, Guiyang, Guizhou Province China; 5 School of Medicine and Health Management, Guizhou Medical University, Guiyang, Guizhou Province China

**Keywords:** Children, Antibiotic use, Appropriateness, Primary care institution

## Abstract

**Background:**

Inappropriate use of antibiotics in children is common in many countries. The purpose of the study was to explore patterns of antibiotic prescribing in children’s outpatient clinics in primary care institutions in a province of southwest China.

**Methods:**

We obtained electronic prescription data from 75 primary care institutions in Guizhou province in 2020. The classification of incorrect spectrum of antibiotics, unnecessary use and combined use of antibiotics was based on the Guiding Principle of Clinical Use of Antibiotics (2015, China) and guidelines from the USA Centers for Disease Control and Prevention. Potential risk factors for inappropriate use of antibiotics were identified using bivariate analyses. The generalized estimation equation was used to identify independent predictors of inappropriate use of antibiotics.

**Results:**

A total of 158,267 antibiotic prescriptions were retrieved. Acute upper respiratory tract infections were the most common diseases, accounting for 74.9% of all prescriptions. The main antibiotic group used was penicillins (63.7%), followed by cephalosporins (18.8%). Of 137,284 visits, 18.3% of antibiotic prescriptions were appropriate and the percentage of unnecessary use, incorrect spectrum of antibiotics and combined use of antibiotics was 76.9, 2.4 and 2.4%, respectively. Physicians with lower professional titles and more than 40 years of work duration were relatively more likely to prescribe inappropriate antibiotics.

**Conclusion:**

The inappropriate use of antibiotics in children is still prominent in primary care institutions of southwest China. The education and training of physicians and caregivers in these institutions should be strengthened.

**Supplementary Information:**

The online version contains supplementary material available at 10.1186/s12875-022-01875-9.

## Background

Antibiotic resistance (ABR) has become a major challenge in the field of global public health [1]. Inappropriate use of antibiotics not only leads to the development of ABR, but also increases various adverse reactions and the financial burden on health services [2]. In recent years, measures to control the inappropriate use of antibiotics have been implemented by health authorities, scientific research and medical institutions in many countries [3–8]. Although the prevalence of overuse and abuse of antibiotics has decreased [9–14], the number of main pathogens of ABR in pediatric patients remain high [15, 16].

The appropriate use of antibiotics in children is critical because there are limited formulations of antibiotics suitable for this population [17, 18]. Studies of antibiotic prescribing patterns among children in primary care institutions in many countries found that inappropriate antibiotic use among children ranged from 19.6 to 79.8% [18–22]. However, children belong to a special drug using group because the organs and functions of their bodies are not fully developed. They also have unique digestive characteristics, lack of liver and kidney metabolism, and incomplete blood-brain barrier function. Antibiotic absorption, distribution, metabolism and excretion are weaker than that in adults [23]. Therefore, more attention should be paid to the inappropriate use of antibiotics in this population.

This study reviewed the antibiotic prescription data of children in outpatient clinics of primary care institutions in Guizhou, southwest China in 2020. The objective of the study was to explore patterns of antibiotic prescribing in children’s outpatient clinics in primary care institutions.

## Materials and methods

### Ethics approval

The study was approved by the Review Committee of Guizhou Medical University (Approval Certificate No. 2019(149)). All participants (physicians) provided written informed consent to participate in the study.

### Study design and setting

A retrospective study was conducted in Guizhou, one of China’s poorest provinces. Antibiotic prescribing patterns for children in primary care institutions were quantified from January to December 2020. The main influencing factors of inappropriate use of antibiotics were explored.

This study has been authorized by the Information Center of Guizhou Provincial Health Commission. A data collection agreement was reached with the Guizhou LianKe Weixin Co., LTD. (LWTC), which developed the electronic Hospital Information System (HIS). The data was obtained through the data port of the information center.

### Data sources

China’s 3-tier hospital system, from top to bottom, includes: tertiary hospitals, secondary hospitals, and primary hospitals. Primary care institutions, which include township health centers and community health service centers, belong to primary hospitals [24]. In 2020, there were 252 public primary care institutions using the HIS system in Guizhou Province. Seventy-five primary care institutions were randomly selected for the study. The two selection criteria were: (1) the institution contained more than three outpatient general practitioners, and (2) the physicians must have been on duty year-round during 2020. Of the one hundred and seventy-two primary care institutions that met the above criteria, 75 primary care institutions were randomly selected by LWTC staff through a random number table. We derived outpatient antibiotic prescription-related information and demographic information of patients from the HIS. The sex, age, education, title and working experience of physicians were provided by the Personnel Management Department of the primary care institutions.

People under 18 years of age who received antibiotics were included in our study, according to the definition of children’s age in *the United Nations Convention on the Rights of the Child* [25] and *Law of the People’s Republic of China on the Protection of Minors* [26]. The classification of diseases was based on *the 10th Edition of the International Classification of Diseases* (ICD-10) [27]. According to *the 2018 National Catalogue for Clinical Application of Antibacterial Drugs* (summarized in Additional file S1), antibiotics were classified into penicillins, cephalosporins, macrolides, quinolones, lincoamides, nitroimidazoles and aminoglycosides. We focused on systemic antibiotics; topical antibiotics such as eye drops and ointments were excluded.

### Appropriate classification of antibiotic use

Our evaluation of the appropriateness of antibiotic prescription was mainly based on the following three aspects: 1) National Health Commission of China for *Guiding Principle of Clinical Use of Antibiotics* introduced in 2015 (Additional file S2), 2) *the United States Centers for Disease Control and Prevention (CDC) Guidelines* for use of antibiotics [28], and 3) based on our previous research [29], we also added the opinions of experts familiar with the situation of domestic primary care institutions. Thus, antibiotic prescriptions in primary care institutions were divided into appropriate and inappropriate use. Appropriate use of antibiotics was further divided into two categories: 1) preferred medication: optimal drug, and 2) antibiotics can be used or substituted: available, not optimal. Inappropriate antibiotic prescribing was further divided into three categories: 1) Unnecessary use: prescribing antibiotics to prevent viral infections, 2) incorrect spectrum of antibiotics: through all kinds of antibiotic drug pharmacological action, antibacterial spectrum, adverse drug reaction and “the empirical treatment can choose antibacterial drugs according to the possible pathogenic bacteria” [17, 28] to judge, e.g., the main pathogens of acute tonsillitis and acute pharyngitis were group A hemolytic streptococcus, and a few were Group C or G hemolytic streptococcus. Therefore, penicillins are preferred medication when antibiotics are treated empirically, and cephalosporins can be considered next. If the patient is allergic to penicillins, quinolones sensitive to hemolytic streptococci can be considered. Lincoamides and macrolides (bacteria such as *Streptococcus pneumoniae* at present are highly resistant to these two antibiotics groups, and are generally not used) also can be used or substituted. Nitroazoles mainly target anaerobic bacteria, and aminoglycosides have poor antibiotic effect on *Streptococcus pneumoniae* group A hemolytic streptococcus. The use of nitroazoles and aminoglycosides in treatment was regarded as incorrect spectrum of antibiotics., and 3) combined use of antibiotics: more than one systemic antibiotic by injection or oral administration at a time by an outpatient physician without any indication, e.g., amoxicillin capsule and ceftazidime injection combined.

### Data analysis

All prescriptions were linked to physicians and patients through coding identification, forming a database of medical service information. A physician can prescribe one or more antibiotics to a patient in a day, but multiple visits per patient per day count as only one visit. Antibiotic prescribing patterns were determined by performing bivariate cross-tabulations between ICD-10 disease classification and antibiotic groups. Potential risk factors for inappropriate use of antibiotics were also identified using a bivariate cross-tabulation. In order to explain the correlation between antibiotic prescriptions prescribed by the same physician and to avoid possible confounding effects of other variables, the generalized estimation equation (GEE) approach was used to identify independent predictors for inappropriate use of antibiotics. All *P*-values were two-sided. R version 4.1.2 was used for all data management and analysis.

## Results

During the study period, 158,267 antibiotics prescribed to 143,809 patients aged 18 years and under were obtained from the HIS. After excluding 823 antibiotic prescriptions labeled in other categories and 2310 topical antibiotic prescriptions, there were 155,134 prescriptions remaining among 143,257 patients. For the purposes of this analysis, only patients who were diagnosed with any of the top 10 common systemic diseases were included in the study, resulting in a total of 150,133 antibiotic prescriptions (96.8%).

**Fig. 1:**
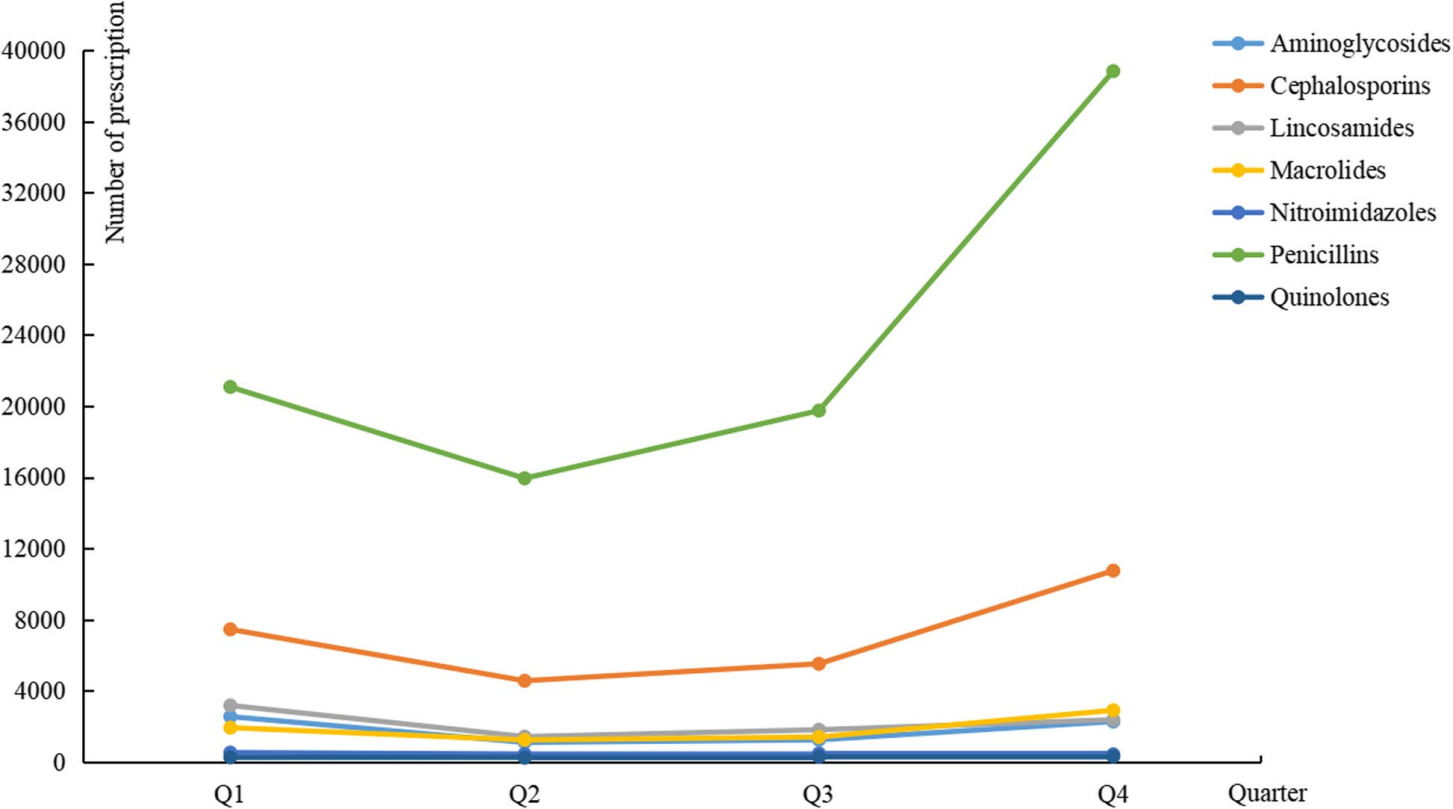
Number of antibiotic prescriptions in each quarter in 2020 stratified by antibiotics classes

Figure 1 shows the number of antibiotic prescriptions per quarter in 2020. Throughout the year, penicillins were prescribed more often than other antibiotics. The second most commonly prescribed antibiotic class was cephalosporins. More penicillins and cephalosporins were prescribed in the first or fourth quarter, while quinolones and nitroimidazoles showed little change in the prescription patterns across the four quarters.

Table 1 shows the distribution of clinical diagnoses, antibiotic group and antibiotic prescriptions. In columns 4–10, “P” represents “Preferred medication”, “A” represents “Antibiotic can be used or substituted”, “I” represents “Incorrect spectrum of antibiotics”, and “U” represents “Unnecessary use”. Diseases of the respiratory system accounted for 86.8% of all antibiotic prescriptions, followed by diseases of the digestive system (6.0%) and diseases of the skin and subcutaneous tissue (2.1%). Penicillins were used the most, accounting for 63.7% of the total prescriptions, followed by cephalosporins (18.8%) and lincosamides (5.8%).

**Table 1:** Distribution of antibiotic prescriptions stratified by clinical diagnosis, antibiotic group and appropriateness of use

ICD 10	Disease	Total	Penicillins	Cephalosporins	Lincosamides	Macrolides	Aminoglycosides	Nitroimidazoles	Quinolones	Appropriate use		Inappropriate use	
										P	A	I	U
**1. Diseases of the respiratory system**		130,371 (86.8)	84,633 (64.9)	24,935 (19.1)	7689 (5.9)	6769 (5.2)	5360 (4.1)	563 (0.4)	422 (0.3)	10,779 (8.3)	12,767 (9.8)	4302 (3.3)	102,523 (78.6)
**Upper respiratory tract infections**		99,794 (76.5)	66,995 (67.1)	17,718 (26.4)	5689 (5.7)	5038 (5.0)	3633 (3.6)	439 (0.4)	282 (0.3)	10,490 (10.5)	6094 (6.1)	3137 (3.1)	80,073 (80.2)
J06	Acute upper respiratory infections of multiple and unspecified sites	76,478 (76.6)	**U** 54,017 (70.6)	**U** 12,078 (22.4)	**U** 4023 (5.3)	**U** 3676 (4.8)	**U** 2221 (2.9)	**U** 220 (0.3)	**U** 243 (0.3)	0 (0.0)	0 (0.0)	0 (0.0)	76,478 (100.0)
J03	Acute tonsillitis	17,849 (17.9)	**P** 9257 (51.9)	**A** 4597 (49.7)	**I** 1485 (8.3)	**A** 991 (5.6)	**I** 1305 (7.3)	**I** 195 (1.1)	**A** 19 (0.1)	9257 (51.9)	5607 (31.4)	2985 (16.7)	0 (0.0)
J39	Other diseases of upper respiratory tract	1756 (1.8)	**U** 1356 (77.2)	**U** 283 (20.9)	**U** 49 (2.8)	**U** 47 (2.7)	**U** 10 (0.6)	**U** 6 (0.3)	**U** 5 (0.3)	0 (0.0)	0 (0.0)	0 (0.0)	1756 (100.0)
J02	Acute pharyngitis	1638 (1.6)	**P** 1023 (62.5)	**A** 335 (32.7)	**I** 85 (5.2)	**A** 134 (8.2)	**I** 49 (3.0)	**I** 8 (0.5)	**A** 4 (0.2)	1023 (62.5)	473 (28.9)	142 (8.7)	0 (0.0)
J00	Acute nasopharyngitis [common cold]	1442 (1.4)	**U** 963 (66.8)	**U** 294 (30.5)	**U** 17 (1.2)	**U** 125 (8.7)	**U** 33 (2.3)	**U** 4 (0.3)	**U** 6 (0.4)	0 (0.0)	0 (0.0)	0 (0.0)	1442 (100.0)
J31	Chronic rhinitis, nasopharyngitis and pharyngitis	397 (0.4)	**U** 255 (64.2)	**U** 59 (23.1)	**U** 16 (4.0)	**U** 51 (12.8)	**U** 6 (1.5)	**U** 5 (1.3)	**U** 5 (1.3)	0 (0.0)	0 (0.0)	0 (0.0)	397 (100.0)
J04	Acute laryngitis and tracheitis	234 (0.2)	**P** 124 (53.0)	**P** 72 (58.1)	**A** 14 (6.0)	**P** 14 (6.0)	**I** 9 (3.8)	**I** 1 (0.4)	**P** 0 (0.0)	210 (89.7)	14 (6.0)	10 (4.3)	0 (0.0)
**Lower respiratory tract infections**		30,577 (23.5)	17,638 (57.7)	7217 (40.9)	2000 (6.5)	1731 (5.7)	1727 (5.6)	124 (0.4)	140 (0.5)	289 (0.9)	6673 (21.8)	1165 (3.8)	22,450 (73.4)
J20	Acute bronchitis	19,766 (64.6)	**U** 11,353 (57.4)	**U** 4918 (43.3)	**U** 1117 (5.7)	**U** 1162 (5.9)	**U** 1066 (5.4)	**U** 61 (0.3)	**U** 89 (0.5)	0 (0.0)	0 (0.0)	0 (0.0)	19,766 (100.0)
J40	Bronchitis, not specified as acute or chronic	6257 (20.5)	**A** 4146 (66.3)	**A** 841 (20.3)	**I** 507 (8.1)	**A** 258 (4.1)	**I** 434 (6.9)	**I** 36 (0.6)	**A** 35 (0.6)	0 (0.0)	5280 (84.4)	977 (15.6)	0 (0.0)
J98	Other respiratory disorders	2122 (6.9)	**U** 1021 (48.1)	**U** 625 (61.2)	**U** 283 (13.3)	**U** 125 (5.9)	**U** 51 (2.4)	**U** 9 (0.4)	**U** 8 (0.4)	0 (0.0)	0 (0.0)	0 (0.0)	2122 (100.0)
J18	Pneumonia, organism unspecified	1554 (5.1)	**A** 566 (36.4)	**A** 667 (42.9)	**I** 51 (3.3)	**A** 142 (9.1)	**I** 117 (7.5)	**I** 8 (0.5)	**A** 3 (0.2)	0 (0.0)	1378 (88.7)	176 (11.3)	0 (0.0)
J21	Acute bronchiolitis	562 (1.8)	**U** 314 (55.9)	**U** 119 (37.9)	**U** 27 (4.8)	**U** 43 (7.7)	**U** 47 (8.4)	**U** 10 (1.8)	**U** 2 (0.4)	0 (0.0)	0 (0.0)	0 (0.0)	562 (100.0)
J22	Unspecified acute lower respiratory infection	316 (1.0)	**P** 238 (75.3)	**P** 47 (14.9)	**A** 15 (4.7)	**P** 1 (0.3)	**I** 12 (3.8)	**I** 0 (0.0)	**P** 3 (0.9)	289 (91.5)	15 (4.7)	12 (3.8)	0 (0.0)
**2. Diseases of the digestive system**		9047 (6.0)	4839 (53.5)	1188 (13.1)	403 (4.5)	307 (3.4)	1280 (14.1)	631 (7.0)	399 (4.4)	2000 (22.1)	1218 (13.5)	388 (4.3)	5441 (60.1)
K52	Other noninfective gastroenteritis and colitis	4360 (48.2)	**U** 2089 (47.9)	**U** 554 (12.7)	**U** 213 (4.9)	**U** 36 (0.8)	**U** 1006 (23.1)	**U** 140 (3.2)	**U** 322 (7.4)	0 (0.0)	0 (0.0)	0 (0.0)	4360 (100.0)
K05	Gingivitis and periodontal diseases	1365 (15.1)	**P** 730 (53.5)	**A** 200 (14.7)	**I** 67 (4.9)	**P** 81 (5.9)	**I** 11 (0.8)	**P** 268 (19.6)	**A** 8 (0.6)	998 (73.1)	289 (21.2)	78 (5.7)	0 (0.0)
K29	Gastritis and duodenitis	1177 (13.0)	**P** 789 (67.0)	**I** 96 (8.2)	**I** 55 (4.7)	**A** 73 (6.2)	**I** 70 (5.9)	**A** 58 (4.9)	**A** 36 (3.1)	789 (67.0)	167 (14.2)	221 (18.8)	0 (0.0)
K30	Functional dyspepsia	776 (8.6)	**U** 488 (62.9)	**U** 106 (13.7)	**U** 5 (1.9)	**U** 22 (2.8)	**U** 121 (15.6)	**U** 0 (0.0)	**U** 24 (3.1)	0 (0.0)	0 (0.0)	0 (0.0)	776 (100.0)
K12	Stomatitis and related lesions	686 (7.6)	**A** 378 (55.1)	**A** 118 (17.2)	**I** 32 (4.7)	**A** 49 (7.1)	**I** 52 (7.6)	**A** 55 (8.0)	**I** 2 (0.3)	0 (0.0)	600 (87.5)	86 (12.5)	0 (0.0)
K04	Diseases of pulp and periapical tissues	218 (2.4)	**P** 103 (47.2)	**A** 30 (13.8)	**A** 3 (1.4)	**A** 23 (10.6)	**I** 1 (0.5)	**P** 58 (26.6)	**A** 0 (0.0)	161 (73.9)	56 (25.7)	1 (0.5)	0 (0.0)
K13	Other diseases of lip and oral mucosa	85 (0.9)	**U** 51 (60.0)	**U** 17 (20.0)	**U** 5 (5.9)	**U** 6 (7.1)	**U** 0 (0.0)	**U** 4 (4.7)	**U** 2 (2.4)	0 (0.0)	0 (0.0)	0 (0.0)	85 (100.0)
K81	Cholecystitis	85 (0.9)	**A** 62 (72.9)	**P** 12 (14.1)	**I** 0 (0.0)	**I** 2 (2.4)	**A** 0 (0.0)	**A** 8 (9.4)	**A** 1 (1.2)	12 (14.1)	71 (83.5)	2 (2.4)	0 (0.0)
K08	Other disorders of teeth and supporting structures	76 (0.8)	**U** 46 (60.5)	**U** 5 (6.6)	**U** 2 (2.6)	**U** 9 (11.8)	**U** 0 (0.0)	**U** 13 (17.1)	**U** 1 (1.3)	0 (0.0)	0 (0.0)	0 (0.0)	76 (100.0)
K35	Acute appendicitis	50 (0.6)	**A** 11 (22.0)	**P** 15 (30.0)	**A** 5 (10.0)	**I** 0 (0.0)	**A** 1 (2.0)	**A** 18 (36.0)	**A** 0 (0.0)	15 (30.0)	35 (70.0)	0 (0.0)	0 (0.0)
K14	Diseases of tongue	48 (0.5)	**U** 32 (66.7)	**U** 3 (6.3)	**U** 1 (2.1)	**U** 5 (10.4)	**U** 7 (14.6)	**U** 0 (0.0)	**U** 0 (0.0)	0 (0.0)	0 (0.0)	0 (0.0)	48 (100.0)
K59	Other functional intestinal disorders	34 (0.4)	**U** 18 (52.9)	**U** 3 (8.8)	**U** 0 (0.0)	**U** 0 (0.0)	**U** 11 (32.4)	**U** 0 (0.0)	**U** 2 (5.9)	0 (0.0)	0 (0.0)	0 (0.0)	34 (100.0)
K11	Diseases of salivary glands	31 (0.3)	**U** 14 (45.2)	**U** 14 (45.2)	**U** 2 (6.5)	**U** 0 (0.0)	**U** 0 (0.0)	**U** 1 (3.2)	**U** 0 (0.0)	0 (0.0)	0 (0.0)	0 (0.0)	31 (100.0)
K92	Other diseases of digestive system	31 (0.3)	**U** 21 (67.7)	**U** 6 (19.4)	**U** 3 (9.7)	**U** 1 (3.2)	**U** 0 (0.0)	**U** 0 (0.0)	**U** 0 (0.0)	0 (0.0)	0 (0.0)	0 (0.0)	31 (100.0)
K36	Other appendicitis	25 (0.3)	**P** 7 (28.0)	**P** 9 (36.0)	**A** 0 (0.0)	**A** 0 (0.0)	**I** 0 (0.0)	**P** 8 (32.0)	**P** 1 (4.0)	25 (100.0)	0 (0.0)	0 (0.0)	0 (0.0)
**3. Diseases of the skin and subcutaneous tissue**		3220 (2.1)	1638 (50.9)	800 (24.8)	297 (9.2)	79 (2.5)	64 (2.0)	321 (10.0)	21 (0.7)	350 (10.9)	18 (0.6)	60 (1.9)	2792 (86.7)
L08	Other local infections of skin and subcutaneous tissue	1556 (48.3)	**U** 727 (46.7)	**U** 434 (27.9)	**U** 87 (5.6)	**U** 25 (1.6)	**U** 34 (2.2)	**U** 231 (14.8)	**U** 18 (1.2)	0 (0.0)	0 (0.0)	0 (0.0)	1556 (100.0)
L23	Allergic contact dermatitis	556 (17.3)	**U** 289 (52.0)	**U** 86 (15.5)	**U** 133 (23.9)	**U** 12 (2.2)	**U** 23 (4.1)	**U** 13 (2.3)	**U** 0 (0.0)	0 (0.0)	0 (0.0)	0 (0.0)	556 (100.0)
L04	Acute lymphadenitis	443 (13.8)	**U** 266 (60.0)	**U** 116 (26.2)	**U** 22 (5.0)	**U** 22 (5.0)	**U** 3 (0.7)	**U** 14 (3.2)	**U** 0 (0.0)	0 (0.0)	0 (0.0)	0 (0.0)	443 (100.0)
L03	Cellulitis	257 (8.0)	**P** 153 (59.5)	**P** 61 (23.7)	**A** 8 (3.1)	**A** 6 (2.3)	**I** 0 (0.0)	**I** 29 (11.3)	**I** 0 (0.0)	214 (83.3)	14 (5.4)	29 (11.3)	0 (0.0)
L02	Cutaneous abscess, furuncle and carbuncle	159 (4.9)	**P** 79 (49.7)	**P** 37 (23.3)	**P** 9 (5.7)	**A** 2 (1.3)	**I** 1 (0.6)	**I** 29 (18.2)	**A** 2 (1.3)	125 (78.6)	4 (2.5)	30 (18.9)	0 (0.0)
L30	Other dermatitis	159 (4.9)	**U** 78 (49.1)	**U** 45 (28.3)	**U** 28 (17.6)	**U** 5 (3.1)	**U** 1 (0.6)	**U** 1 (0.6)	**U** 1 (0.6)	0 (0.0)	0 (0.0)	0 (0.0)	159 (100.0)
L24	Irritant contact dermatitis	32 (1.0)	**U** 15 (46.9)	**U** 8 (25.0)	**U** 6 (18.8)	**U** 2 (6.3)	**U** 0 (0.0)	**U** 1 (3.1)	**U** 0 (0.0)	0 (0.0)	0 (0.0)	0 (0.0)	32 (100.0)
L50	Urticaria	29 (0.9)	**U** 16 (55.2)	**U** 8 (27.6)	**U** 3 (10.3)	**U** 0 (0.0)	**U** 2 (6.9)	**U** 0 (0.0)	**U** 0 (0.0)	0 (0.0)	0 (0.0)	0 (0.0)	29 (100.0)
L70	Acne	17 (0.5)	**U** 7 (41.2)	**U** 2 (11.8)	**U** 1 (5.9)	**U** 5 (29.4)	**U** 0 (0.0)	**U** 2 (11.8)	**U** 0 (0.0)	0 (0.0)	0 (0.0)	0 (0.0)	17 (100.0)
L01	Impetigo	12 (0.4)	**P** 8 (66.7)	**P** 3 (25.0)	**P** 0 (0.0)	**A** 0 (0.0)	**I** 0 (0.0)	**I** 1 (8.3)	**A** 0 (0.0)	11 (91.7)	0 (0.0)	1 (8.3)	0 (0.0)
**4. Symptoms, signs and abnormal clinical and laboratory findings not elsewhere classified**		2609 (1.7)	1590 (60.9)	376 (14.4)	145 (5.6)	94 (3.6)	257 (9.9)	59 (2.3)	88 (3.4)	0 (0.0)	0 (0.0)	0 (0.0)	2609 (100.0)
R10	Abdominal and pelvic pain	1827 (70.0)	**U** 1132 (62.0)	**U** 260 (14.2)	**U** 58 (3.2)	**U** 47 (2.6)	**U** 204 (11.2)	**U** 46 (2.5)	**U** 80 (4.4)	0 (0.0)	0 (0.0)	0 (0.0)	1827 (100.0)
R05	Cough	161 (6.2)	**U** 91 (56.5)	**U** 18 (11.2)	**U** 12 (7.5)	**U** 7 (4.3)	**U** 29 (18.0)	**U** 0 (0.0)	**U** 4 (2.5)	0 (0.0)	0 (0.0)	0 (0.0)	161 (100.0)
R59	Enlarged lymph nodes	146 (5.6)	**U** 104 (71.2)	**U** 20 (13.7)	**U** 9 (6.2)	**U** 5 (3.4)	**U** 4 (2.7)	**U** 4 (2.7)	**U** 0 (0.0)	0 (0.0)	0 (0.0)	0 (0.0)	146 (100.0)
R50	Fever of other and unknown origin	140 (5.4)	**U** 65 (46.4)	**U** 19 (13.6)	**U** 47 (33.6)	**U** 2 (1.4)	**U** 4 (2.9)	**U** 1 (0.7)	**U** 2 (1.4)	0 (0.0)	0 (0.0)	0 (0.0)	140 (100.0)
R04	Haemorrhage from respiratory passages	93 (3.6)	**U** 60 (64.5)	**U** 13 (14.0)	**U** 7 (7.5)	**U** 12 (12.9)	**U** 0 (0.0)	**U** 1 (1.1)	**U** 0 (0.0)	0 (0.0)	0 (0.0)	0 (0.0)	93 (100.0)
R07	Pain in throat and chest	70 (2.7)	**U** 51 (72.9)	**U** 8 (11.4)	**U** 3 (4.3)	**U** 5 (7.1)	**U** 3 (4.3)	**U** 0 (0.0)	**U** 0 (0.0)	0 (0.0)	0 (0.0)	0 (0.0)	70 (100.0)
R51	Headache	63 (2.4)	**U** 25 (39.7)	**U** 13 (20.6)	**U** 8 (12.7)	**U** 12 (19.0)	**U** 0 (0.0)	**U** 3 (4.8)	**U** 2 (3.2)	0 (0.0)	0 (0.0)	0 (0.0)	63 (100.0)
R22	Localized swelling, mass and lump of skin and subcutaneous tissue	42 (1.6)	**U** 27 (64.3)	**U** 12 (28.6)	**U** 0 (0.0)	**U** 1 (2.4)	**U** 0 (0.0)	**U** 2 (4.8)	**U** 0 (0.0)	0 (0.0)	0 (0.0)	0 (0.0)	42 (100.0)
R21	Rash and other nonspecific skin eruption	38 (1.5)	**U** 21 (55.3)	**U** 10 (26.3)	**U** 1 (2.6)	**U** 3 (7.9)	**U** 1 (2.6)	**U** 2 (5.3)	**U** 0 (0.0)	0 (0.0)	0 (0.0)	0 (0.0)	38 (100.0)
R11	Nausea and vomiting	29 (1.1)	**U** 14 (48.3)	**U** 3 (10.3)	**U** 0 (0.0)	**U** 0 (0.0)	U 12 (41.4)	**U** 0 (0.0)	**U** 0 (0.0)	0 (0.0)	0 (0.0)	0 (0.0)	29 (100.0)
**5. Injury, poisoning and certain other consequences of external causes**		1685 (1.1)	1127 (66.9)	315 (18.7)	77 (4.6)	37 (2.2)	21 (1.2)	100 (5.9)	8 (0.5)	723 (42.9)	73 (4.3)	15 (0.9)	874 (51.9)
T14	Injury of unspecified body region	748 (44.4)	**U** 482 (64.4)	**U** 120 (16.0)	**U** 39 (5.2)	**U** 20 (2.7)	**U** 21 (2.8)	**U** 61 (8.2)	**U** 5 (0.7)	0 (0.0)	0 (0.0)	0 (0.0)	748 (100.0)
S01	Open wound of head	576 (34.2)	**P** 406 (70.5)	**P** 114 (19.8)	**A** 22 (3.8)	**I** 10 (1.7)	**I** 0 (0.0)	**A** 24 (4.2)	**A** 0 (0.0)	520 (90.3)	46 (8.0)	10 (1.7)	0 (0.0)
S00	Superficial injury of head	177 (10.5)	**P** 137 (77.4)	**P** 27 (15.3)	**A** 6 (3.4)	**A** 3 (1.7)	**I** 0 (0.0)	**A** 4 (2.3)	**A** 0 (0.0)	164 (92.7)	13 (7.3)	0 (0.0)	0 (0.0)
T11	Other injuries of upper limb, level unspecified	126 (7.5)	**U** 63 (50.0)	**U** 40 (31.7)	**U** 10 (7.9)	**U** 4 (3.2)	**U** 0 (0.0)	**U** 6 (4.8)	**U** 3 (2.4)	0 (0.0)	0 (0.0)	0 (0.0)	126 (100.0)
T13	Other injuries of lower limb, level unspecified	58 (3.4)	**P** 39 (67.2)	**A** 14 (24.1)	**A** 0 (0.0)	**A** 0 (0.0)	**I** 0 (0.0)	**I** 5 (8.6)	**A** 0 (0.0)	39 (67.2)	14 (24.1)	5 (8.6)	0 (0.0)
**6. Diseases of the genitourinary system**		891 (0.6)	376 (42.2)	232 (26.0)	45 (5.1)	30 (3.4)	13 (1.5)	108 (12.1)	87 (9.8)	202 (22.7)	151 (16.9)	41 (4.6)	497 (55.8)
N39	Other disorders of urinary system	359 (40.3)	**U** 155 (43.2)	**U** 101 (28.1)	**U** 6 (1.7)	**U** 11 (3.1)	**U** 10 (2.8)	**U** 26 (7.2)	**U** 50 (13.9)	0 (0.0)	0 (0.0)	0 (0.0)	359 (100.0)
N34	Urethritis and urethral syndrome	194 (21.8)	**P** 103 (53.1)	**P** 48 (24.7)	**I** 1 (0.5)	**P** 9 (4.6)	**I** 2 (1.0)	**I** 9 (4.6)	**A** 22 (11.3)	160 (82.5)	22 (11.3)	12 (6.2)	0 (0.0)
N48	Other disorders of penis	138 (15.5)	**U** 66 (47.8)	**U** 37 (26.8)	**U** 10 (7.2)	**U** 6 (4.3)	**U** 1 (0.7)	**U** 16 (11.6)	**U** 2 (1.4)	0 (0.0)	0 (0.0)	0 (0.0)	138 (100.0)
N73	Other female pelvic inflammatory diseases	116 (13.0)	**A** 34 (29.3)	**P** 24 (20.7)	**I** 8 (6.9)	**A** 3 (2.6)	**I** 0 (0.0)	**A** 36 (31.0)	**A** 11 (9.5)	24 (20.7)	84 (72.4)	8 (6.9)	0 (0.0)
N47	Redundant prepuce, phimosis and paraphimosis	84 (9.4)	**P** 18 (21.4)	**A** 22 (26.2)	**A** 20 (23.8)	**A** 1 (1.2)	**I** 0 (0.0)	**I** 21 (25.0)	**A** 2 (2.4)	18 (21.4)	45 (53.6)	21 (25.0)	0 (0.0)
**7. Diseases of the circulatory system**		669 (0.4)	416 (62.2)	150 (22.4)	33 (4.9)	29 (4.3)	14 (2.1)	21 (3.1)	6 (0.9)	20 (3.0)	1 (0.1)	8 (1.2)	640 (95.7)
I88	Nonspecific lymphadenitis	589 (88.0)	**U** 364 (61.8)	**U** 138 (23.4)	**U** 30 (5.1)	**U** 27 (4.6)	**U** 14 (2.4)	**U** 15 (2.5)	**U** 1 (0.2)	0 (0.0)	0 (0.0)	0 (0.0)	589 (100.0)
I84	Haemorrhoids	42 (6.3)	**U** 26 (61.9)	**U** 4 (9.5)	**U** 2 (4.8)	**U** 2 (4.8)	**U** 0 (0.0)	**U** 5 (11.9)	**U** 3 (7.1)	0 (0.0)	0 (0.0)	0 (0.0)	42 (100.0)
I00	Rheumatic fever without mention of heart involvement	21 (3.1)	**P** 18 (85.7)	**A** 1 (4.8)	**I** 1 (4.8)	**A** 0 (0.0)	**I** 0 (0.0)	**I** 1 (4.8)	**I** 0 (0.0)	18 (85.7)	1 (4.8)	2 (9.5)	0 (0.0)
I67	Other cerebrovascular diseases	9 (1.3)	**U** 6 (66.7)	**U** 2 (22.2)	**U** 0 (0.0)	**U** 0 (0.0)	**U** 0 (0.0)	**U** 0 (0.0)	**U** 1 (11.1)	0 (0.0)	0 (0.0)	0 (0.0)	9 (100.0)
I40	Acute myocarditis	8 (1.2)	**P** 2 (25.0)	**I** 5 (62.5)	**I** 0 (0.0)	**I** 0 (0.0)	**I** 0 (0.0)	**I** 0 (0.0)	**I** 1 (12.5)	2 (25.0)	0 (0.0)	6 (75.0)	0 (0.0)
**8. Diseases of the ear and mastoid process**		603 (0.4)	384 (63.7)	127 (21.1)	16 (2.7)	33 (5.5)	9 (1.5)	29 (4.8)	5 (0.8)	410 (68.0)	80 (13.3)	37 (6.1)	76 (12.6)
H66	Suppurative and unspecified otitis media	468 (77.6)	**P** 290 (62.0)	**P** 105 (22.4)	**A** 11 (2.4)	**A** 23 (4.9)	**I** 7 (1.5)	**I** 27 (5.8)	**A** 5 (1.1)	395 (84.4)	39 (8.3)	34 (7.3)	0 (0.0)
H65	Nonsuppurative otitis media	44 (7.3)	**U** 32 (72.7)	**U** 6 (13.6)	**U** 3 (6.8)	**U** 3 (6.8)	**U** 0 (0.0)	**U** 0 (0.0)	**U** 0 (0.0)	0 (0.0)	0 (0.0)	0 (0.0)	44 (100.0)
H60	Otitis externa	43 (7.1)	**A** 28 (65.1)	**A** 9 (20.9)	**A** 1 (2.3)	**P** 3 (7.0)	**I** 2 (4.7)	**A** 0 (0.0)	**A** 0 (0.0)	3 (7.0)	38 (88.4)	2 (4.7)	0 (0.0)
H61	Other disorders of external ear	32 (5.3)	**U** 25 (78.1)	**U** 4 (12.5)	**U** 0 (0.0)	**U** 2 (6.3)	**U** 0 (0.0)	**U** 1 (3.1)	**U** 0 (0.0)	0 (0.0)	0 (0.0)	0 (0.0)	32 (100.0)
H70	Mastoiditis and related conditions	16 (2.7)	**P** 9 (56.3)	**P** 3 (18.8)	**A** 1 (6.3)	**A** 2 (12.5)	**I** 0 (0.0)	**I** 1 (6.3)	**A** 0 (0.0)	12 (75.0)	3 (18.8)	1 (6.3)	0 (0.0)
**9. Certain infectious and parasitic diseases**		590 (0.4)	329 (55.8)	73 (12.4)	40 (6.8)	4 (0.7)	120 (20.3)	9 (1.5)	15 (2.5)	1 (0.2)	185 (31.4)	78 (13.2)	326 (55.3)
A09	Other gastroenteritis and colitis of infectious and unspecified origin	207 (35.1)	**A** 145 (70.0)	**I** 15 (7.2)	**I** 0 (0.0)	**I** 2 (1.0)	**I** 35 (16.9)	**I** 2 (1.0)	**I** 8 (3.9)	0 (0.0)	145 (70.0)	62 (30.0)	0 (0.0)
B00	Herpesviral [herpes simplex] infections	137 (23.2)	**U** 59 (43.1)	**U** 25 (18.2)	**U** 39 (28.5)	**U** 0 (0.0)	**U** 11 (8.0)	**U** 3 (2.2)	**U** 0 (0.0)	0 (0.0)	0 (0.0)	0 (0.0)	137 (100.0)
A08	Viral and other specified intestinal infections	101 (17.1)	**U** 40 (39.6)	**U** 6 (5.9)	**U** 0 (0.0)	**U** 1 (1.0)	**U** 44 (43.6)	**U** 4 (4.0)	**U** 6 (5.9)	0 (0.0)	0 (0.0)	0 (0.0)	101 (100.0)
B08	Other viral infections characterized by skin and mucous membrane lesions, not elsewhere classified	88 (14.9)	**U** 52 (59.1)	**U** 21 (23.9)	**U** 0 (0.0)	**U** 0 (0.0)	**U** 15 (17.0)	**U** 0 (0.0)	**U** 0 (0.0)	0 (0.0)	0 (0.0)	0 (0.0)	88 (100.0)
A04	Other bacterial intestinal infections	57 (9.7)	**A** 33 (57.9)	**A** 6 (10.5)	**I** 1 (1.8)	**A** 1 (1.8)	**I** 15 (26.3)	**I** 0 (0.0)	**P** 1 (1.8)	1 (1.8)	40 (70.2)	16 (28.1)	0 (0.0)
**10.Diseases of the eye and adnexa**		448 (0.3)	272 (60.7)	76 (17.0)	18 (4.0)	64 (14.3)	3 (0.7)	12 (2.7)	3 (0.7)	37 (8.3)	207 (46.2)	166 (37.1)	38 (8.5)
H16	Keratitis	135 (30.1)	**I** 85 (63.0)	**I** 27 (20.0)	**I** 6 (4.4)	**I** 13 (9.6)	**A** 1 (0.7)	**I** 1 (0.7)	**P** 2 (1.5)	2 (1.5)	1 (0.7)	132 (97.8)	0 (0.0)
H01	Other inflammation of eyelid	128 (28.6)	**A** 86 (67.2)	**I** 23 (18.0)	**A** 3 (2.3)	**A** 13 (10.2)	**A** 1 (0.8)	**I** 2 (1.6)	**A** 0 (0.0)	0 (0.0)	103 (80.5)	25 (19.5)	0 (0.0)
H00	Hordeolum and chalazion	88 (19.6)	**A** 52 (59.1)	**A** 14 (15.9)	**P** 3 (3.4)	**P** 11 (12.5)	**P** 0 (0.0)	**I** 8 (9.1)	**P** 0 (0.0)	14 (15.9)	66 (75.0)	8 (9.1)	0 (0.0)
H10	Conjunctivitis	59 (13.2)	**A** 29 (49.2)	**A** 8 (13.6)	**P** 5 (8.5)	**P** 14 (23.7)	**P** 1 (1.7)	**I** 1 (1.7)	**P** 1 (1.7)	21 (35.6)	37 (62.7)	1 (1.7)	0 (0.0)
H02	Other disorders of eyelid	38 (8.5)	**U** 20 (52.6)	**U** 4 (10.5)	**U** 1 (2.6)	**U** 13 (34.2)	**U** 0 (0.0)	**U** 0 (0.0)	**U** 0 (0.0)	0 (0.0)	0 (0.0)	0 (0.0)	38 (100.0)
**Total prescriptions**		150,133	95,604 (63.7)	28,272 (18.8)	8763 (5.8)	7446 (5.0)	7141 (4.8)	1853 (1.2)	1054 (0.7)	14,522 (9.7)	14,700 (9.8)	5095 (3.4)	115,816 (77.1)

Numbers in the table are frequency (%)

P: Preferred medication

A: Antibiotic can be used or substituted

I: Incorrect spectrum of antibiotics

U: Unnecessary use

The highest rate of inappropriate use was seen for symptoms, signs and abnormal clinical and laboratory finding not elsewhere classified (100%), diseases of the circulatory system (96.9%), and diseases of the skin and subcutaneous tissue (88.6%). Incorrect spectrum of antibiotic was common for children with diseases of the eye and adnexa (37.1%), certain infectious and parasitic diseases (13.2%), and diseases of the ear and mastoid process (6.1%). The unnecessary use of antibiotics for diseases of the ear and mastoid process and diseases of the eye and adnexa was 12.6% and 8.5%, respectively. Unnecessary use of antibiotics for the other eight systemic diseases exceeded 50%. Diseases of the ear and mastoid process (81.3%), diseases of the eye and adnexa (54.5%) and injury, poisoning and certain other consequences of external causes (47.2%) were the top three ranked diseases in terms of appropriateness of antibiotics use.

Table 2 compares the distribution of patterns of antibiotics use by physicians’ and patients-related factors. In column 5, “Combined use of antibiotics” refers to when a physician prescribes two or more groups of antibiotics for the same patient in the same visit. The percentage of different antibiotic prescription types decreased compared to Table 1 as two or more antibiotics prescribed to the same patient on the same day were considered a single visit. As shown in Table 2, the proportions of prescriptions that were “preferred medication”, “antibiotic can be used or substituted”, “combined use of antibiotics”, “incorrect spectrum of antibiotics” and “unnecessary use” were 9.5%, 8.8%, 2.4%, 2.4% and 76.9%, respectively. Bivariate analysis showed that all variables were statistically significant (all *P* <  0.001). Therefore, all variables were included in the multivariate analysis.

**Table 2:** Factors associated with inappropriate use of antibiotics on bivariate analysis

Characteristic	Total, N (%)	Appropriate use, n (%)		Inappropriate use, n (%)			Chi-square test	
		Preferred medication	Antibiotic can be used or substituted	Combined use of antibiotics	Incorrect spectrum of antibiotics	Unnecessary use	χ^2^	*P* value
**Total**	137,284	12,991 (9.5)	12,036 (8.8)	3361 (2.4)	3308 (2.4)	105,588 (76.9)		
**Physician-related factors**								
**Sex**							256.80	< 0.001
Female	43,202 (31.5)	4642 (10.7)	3247 (7.5)	1041 (2.4)	887 (2.1)	33,385 (77.3)		
Male	94,082 (68.5)	8349 (8.9)	8789 (9.3)	2320 (2.5)	2421 (2.6)	72,203 (76.7)		
**Age group (years)**							523.01	< 0.001
23–32	48,348 (35.2)	5042 (10.4)	4043 (8.4)	1418 (2.9)	1126 (2.3)	36,719 (75.9)		
33–40	46,024 (33.5)	4390 (9.5)	3510 (7.6)	930 (2.0)	921 (2.0)	36,273 (78.8)		
41–65	42,912 (31.3)	3559 (8.3)	4483 (10.4)	1013 (2.4)	1261 (2.9)	32,596 (76.0)		
**Professional title**							146.91	< 0.001
Associate chief physician	5601 (4.1)	646 (11.5)	535 (9.6)	82 (1.5)	163 (2.9)	4175 (74.5)		
Attending physician	16,559 (12.1)	1496 (9.0)	1284 (7.8)	291 (1.8)	353 (2.1)	13,135 (79.3)		
Resident physician	115,124 (83.9)	10,849 (9.4)	10,217 (8.9)	2988 (2.6)	2792 (2.4)	88,278 (76.7)		
**Education**							447.37	< 0.001
College	57,121 (41.6)	5907 (10.3)	4697 (8.2)	1231 (2.2)	1031 (1.8)	44,255 (77.5)		
Junior college	57,979 (42.2)	5333 (9.2)	5607 (9.7)	1549 (2.7)	1518 (2.6)	43,972 (75.8)		
Technical secondary school	22,184 (16.2)	1751 (7.9)	1732 (7.8)	581 (2.6)	759 (3.4)	17,361 (78.3)		
**Work duration (years)**							2466.86	< 0.001
≤ 5	30,322 (22.1)	3046 (10.0)	2925 (9.6)	1014 (3.3)	961 (3.2)	22,376 (73.8)		
6–10	50,214 (36.6)	4906 (9.8)	2966 (5.9)	903 (1.8)	602 (1.2)	40,837 (81.3)		
11–20	22,207 (16.2)	2225 (10.0)	2181 (9.8)	579 (2.6)	592 (2.7)	16,630 (74.9)		
21–30	21,077 (15.4)	1782 (8.5)	2338 (11.1)	489 (2.3)	864 (4.1)	15,604 (74.0)		
31–39	10,364 (7.5)	840 (8.1)	1521 (14.7)	354 (3.4)	236 (2.3)	7413 (71.5)		
≥ 40	3100 (2.3)	192 (6.2)	105 (3.4)	22 (0.7)	53 (1.7)	2728 (88.0)		
**Patient-related factors**								
**Sex**							88.17	< 0.001
Female	63,023 (45.9)	5659 (9.0)	5399 (8.6)	1439 (2.3)	1379 (2.2)	49,147 (78.0)		
Male	74,261 (54.1)	7332 (9.9)	6637 (8.9)	1922 (2.6)	1929 (2.6)	56,441 (76.0)		
**Age group (years)**							475.07	< 0.001
[0,1]	8417 (6.1)	445 (5.3)	775 (9.2)	251 (3.0)	265 (3.1)	6681 (79.4)		
(1,2]	13,806 (10.1)	989 (7.2)	1143 (8.3)	330 (2.4)	359 (2.6)	10,985 (79.6)		
(2,5]	38,485 (28.0)	3486 (9.1)	3582 (9.3)	875 (2.3)	957 (2.5)	29,585 (76.9)		
(5,11]	46,863 (34.1)	4894 (10.4)	4257 (9.1)	1142 (2.4)	1014 (2.2)	35,556 (75.9)		
(11,18]	29,713 (21.6)	3177 (10.7)	2279 (7.7)	763 (2.6)	713 (2.4)	22,781 (76.7)		
**Quarter**^a^							1312.36	< 0.001
Q1	32,611 (23.8)	3194 (9.8)	3489 (10.7)	1185 (3.6)	1293 (4.0)	23,450 (71.9)		
Q2	22,901 (16.7)	2249 (9.8)	1833 (8.0)	510 (2.2)	489 (2.1)	17,820 (77.8)		
Q3	28,002 (20.4)	3050 (10.9)	2542 (9.1)	614 (2.2)	697 (2.5)	21,099 (75.3)		
Q4	53,770 (39.2)	4498 (8.4)	4172 (7.8)	1052 (2.0)	829 (1.5)	43,219 (80.4)		
**Antibiotic route**							20,893.03	< 0.001
Injection	25,099 (18.3)	1442 (5.7)	3272 (13.0)	2499 (10.0)	2989 (11.9)	14,897 (59.4)		
Oral	112,185 (81.7)	11,549 (10.3)	8764 (7.8)	862 (0.8)	319 (0.3)	90,691 (80.8)		
**Insurance**							1006.17	< 0.001
Fully out-of-pocket	18,394 (13.4)	1952(10.6)	1982 (10.8)	782 (4.3)	860 (4.7)	12,818 (69.7)		
New rural cooperative medical system	118,890 (86.6)	11,039 (9.3)	10,054 (8.5)	2579 (2.2)	2448 (2.1)	92,770 (78.0)		

^a^Quarters

Q1: January–March

Q2: April–June

Q3: July–September

Q4: October – December

Table 3 shows factors associated with inappropriate antibiotic use on multivariate analysis. As shown in Table 3, for physician-related factors, being male, older than 32 years, having a lower professional title, and having a lower level of education were associated with a higher likelihood of inappropriate antibiotic use. In terms of work duration, we found that physicians with 6 to 10 years of service and those with more than 40 years of work duration were more likely to prescribe inappropriate antibiotics. For patient-related factors, females and those aged 0–1 years had a higher likelihood of being prescribed inappropriate antibiotics. Physicians were more likely to prescribe inappropriate antibiotics in quarters 3 and 4 compared to the first quarter of 2020. Finally, children insured with the New Rural Cooperative Medical system were more likely to be prescribed inappropriate antibiotics than those who fully paid for them out-of-pocket.

**Table 3:** Factors predicting inappropriate use of antibiotics on multivariate analysis

Characteristic	Adjusted OR (95% CI)	*P* value
**Physician-related factors Sex: ref = Female**		
Male	1.08 (1.04, 1.11)	<0.001
**Age: ref = 23–32 years**		
33–40	1.17 (1.12, 1.22)	<0.001
41–65	1.38 (1.28, 1.49)	<0.001
**Professional title: ref = Associate chief physician**		
Attending physician	1.83 (1.68, 2.00)	<0.001
Resident physician	1.42 (1.32, 1.54)	<0.001
**Education: ref = College**		
Junior college	1.08 (1.05, 1.12)	<0.001
Technical secondary school	1.49 (1.42, 1.57)	<0.001
**Work duration: ref = ≤5 (years)**		
6–10	1.31 (1.26, 1.37)	<0.001
11–20	0.82 (0.77, 0.87)	<0.001
21–30	0.73 (0.67,0.80)	<0.001
31–39	0.53 (0.49, 0.58)	<0.001
≥ 40	1.63 (1.40, 1.90)	<0.001
**Patient-related factors Sex: ref = Female**		
Male	0.92 (0.90, 0.95)	<0.001
**Age: ref [0,1] (years)**		
(1,2]	0.88 (0.81, 0.94)	<0.001
(2,5]	0.70 (0.65, 0.75)	<0.001
(5,11]	0.64 (0.60, 0.68)	<0.001
(11,18]	0.69 (0.64, 0.74)	<0.001
**Quarter**^**a**^**: ref Q1**		
Q2	1.00 (0.96, 1.05)	0.8496
Q3	1.10 (1.06, 1.15)	<0.001
Q4	1.08 (1.05, 1.13)	<0.001
**Route: ref = Injection**		
Oral	0.96 (0.93, 1.00)	0.0614
**Insurance: ref = Fully out-of-pocket**		
New rural cooperative medical system	1.32 (1.27, 1.38)	<0.001

*OR* Odds ratio, *CI* Confidence interval, *Ref* Reference group

^a^Quarter

Q1: January–March

Q2: April–June

Q3: July–September

Q4: October–December

## Discussion

In this retrospective study, 75 primary care institutions in Guizhou Province were selected to describe the prescription patterns of antibacterial drugs among children in 2020. Overall, the rate of antibiotic prescriptions was highest in the fourth quarter, followed by the first quarter. Among the antibiotics prescribed, penicillins and cephalosporins were the most used antibiotics groups. The most common childhood diseases were the diseases of the respiratory system (86.8%), followed by diseases of the digestive system (6.0%) and diseases of the skin and subcutaneous tissue (2.1%). Overall, 80.5% of antibiotic prescriptions were inappropriate. Physicians with lower professional titles and more than 40 years of work duration were relatively more likely to prescribe inappropriate antibiotics.

In this study, *acute upper respiratory infections of multiple and unspecified sites* (J06, 58.7%) and *acute tonsillitis* (J03, 13.7%) were the most common upper respiratory diseases for which antibiotics were administered in children, accounting for 72.4%. A study from China reported an antibiotic prescription rate for acute upper respiratory tract infections in children of 77.6% while in other countries reported rates ranged from 28.7% (Japan) - 76.2% (Albania) [30–34]. However, the Guidelines for the Clinical Application of Antibiotics in China [17], Practical Diagnosis and Treatment of Pediatric Diseases [35] and the United States CDC [28] state that acute upper respiratory tract infections are the most common community-acquired infections, most commonly caused by viruses such as rhinoviruses, coronaviruses, and influenza viruses. The course of disease is generally self-limited and does not require antibiotic treatment. Treatment of symptoms if often the best form of care, especially in children, and symptoms usually subside within a few days.

Therefore, in Table 1, J06 (*Acute upper respiratory infections of multiple and unspecified sites*) does involve prescription of all antibiotics need not be used (U: unnecessary use). For J03 (*Acute tonsillitis*), penicillin is preferred in more severe cases where bacterial infection is suspected (e.g., tonsillitis caused by streptococcus) (P: preferred medication). Cephalosporins, macrolides, and quinolones also can be used. If lincosamides, aminoglycosides and nitroimidazoles were used, the antibiotic spectrum is incorrect (I: incorrect spectrum of antibiotics).

Lower respiratory tract infections, which include *acute bronchitis* (15.2%) and *bronchitis, not specified as acute or chronic* (4.8%), was the second most common childhood disease class in our study. Studies in China [36], Japan [31] and France [37] found that antibiotic prescription rates for bronchitis were 10.9%, 11.9% and 14.6%, respectively. However, for bronchitis, it should be clear whether it is viral infection or bacterial infection. Viral infection without antibiotics. Suspected bacterial infection can be used penicillin intramuscular injection or oral cephalosporin [35]. Therefore, in Table 1, J20 (*Acute bronchitis*) shows that all antibiotics need not be used (U: unnecessary use). For J40 (*Bronchitis, not specified as acute or chronic*), penicillins, cephalosporins, macrolides and quinolones shown antibiotic can be used or substituted (A: antibiotic can be used or substituted). If lincosamides, aminoglycoside, nitroimidazole were used, the antibiotic spectrum is incorrect (I: incorrect spectrum of antibiotics).

The use of antibiotics for *acute upper respiratory tract infections of multiple and unspecified sites* (J06) or *acute bronchitis* (J20) is inappropriate because it may trigger allergies, infections, and even endanger the child’s life [38]. This scenario likely to lead to ABR in children. The use of antibiotics in children is more likely to kill susceptible strains, leading to proliferation of resistant strains and replacement of susceptible strains, resulting in a sharp increase in drug resistance of bacteria [39].

In this study, the number of children diagnosed with acute otitis media and urinary tract infections was lower than other studies [37, 40]. Although otitis media usually occurs in children, otoscopy is necessary for a definitive diagnosis. Primary care institutions have the lowest testing capacity in China. They have no instrument to examine the inner ear canal. Most primary physicians also have no expertise in otorhinolaryngology. Patients with ear, nose and throat problems are referred to superior hospitals. In addition, in this study, we only analyzed systemic antibiotic prescriptions, excluding local antibiotic prescriptions such as ear drops. This may indirectly lead to low prescriptions for otitis media. For urinary tract infection, most primary care institutions are general outpatient departments, and primary physicians do not have professional knowledge of urinary system diagnosis and treatment. If a child develops symptoms of urinary tract infection, most patients will go to the specialized outpatient clinic of a superior hospital.

Overall, the unnecessary use of antibiotics occurred in all 10 systemic disease classifications in the study, accounting for 63.6% of all antibiotic prescriptions. This included diseases of the respiratory system (J06, J39, J00, J31, J20, J98, J21), diseases of the digestive system (K52, K30, K13, K08, K14, K59, K11, K92), diseases of the skin and subcutaneous tissue (L08, L23, L04, L30, L24, L50, L70), symptoms, signs and abnormal clinical and laboratory findings not elsewhere classified (R10, R05, R59, R50, R04, R07, R51, R22, R21, R11), injury, poisoning and certain other consequences of external causes (T14, T11), diseases of the genitourinary system (N39, N48), diseases of the circulatory system (I88, I84, I67), diseases of the ear and mastoid process (H65, H61), certain infectious and parasitic diseases (B00, A08, B08), and diseases of the eye and adnexa (H02). It should be noted that the 10 sub-diseases under *symptoms, signs and abnormal clinical and laboratory findings not elsewhere classified* (R10, R05, R59, R50, R04, R07, R51, R22, R21, R11) were all diseases for which antibiotics are unnecessary. The rate of inappropriate antibiotics use for these diseases often reached 100%. When treating such childhood diseases, physicians should make specific clinical diagnoses based on typical signs and symptoms [17, 28]. It is particularly important to stress that when physicians suspect a child has severe pneumonia according to typical signs and symptoms, the child should be transferred to a superior hospital in a timely manner [41]. According to National Health Commission of China for Guiding Principle of Clinical Use of Antibiotics introduced in 2015, there is a very limited range of antibiotics suitable for use by those aged 18 years and under [17]. Physicians should be more cautious about prescribing antibiotics for children as widespread use could exacerbate ABR.

We also found from those antibiotic prescriptions with incorrect spectrum of antibiotics accounted for 2.4% of all antibiotic prescriptions. Except for *the systemic disease of symptoms, signs and abnormal clinical and laboratory findings not elsewhere classified*, the other 9 systemic diseases all had the condition of incorrect spectrum of antibiotics. *Diseases of the eye and adnexa* had the highest proportion of incorrect spectrum of antibiotics (37.0%). The proportion of incorrect spectrum of antibiotics was highest in the *sub-disease (keratitis) of diseases of the eye and adnexa* (97.8%). Penicillins, cephalosporins, macrolides, lincosamides and nitroimidazoles are the incorrect spectrum of antibiotics for these particular diseases. According to *Ophthalmology Clinical Guidelines of American Academy of Ophthalmology (2nd edition)* [42], *Ophthalmology of China (9th edition)* [43] and *National Health Commission of China for Guiding Principle of Clinical Use of Antibiotics introduced in 2015* [17], quinolones and aminoglycosides are the preferred medication treatment of *keratitis* treatment, especially in children.

In our study, “Incorrect spectrum of antibiotics” (2.4%) and “Combined use of antibiotics” (2.4%) were the two types of inappropriate antibiotics use that accounted for the least proportion. The proportion of “Incorrect spectrum of antibiotics” in this study was low, mainly because the proportion was distributed differently among different diseases. For example, the proportion for keratitis and acute myocarditis was more than 75% of cases. The low inappropriate rate of “Combined use of antibiotics” was primarily due to the absence of diseases such as tuberculosis, leprosy or other diseases requiring combination therapy in primary care institutions [29]. In addition, we used a very strict definition of drug combinations: more than one systemic antibiotic by injection or oral administration at a time by an outpatient physician without any indication. Previous studies [44–46] have raised the issue of antibiotic combinations. Therefore, even if the numbers are low, “Incorrect spectrum of antibiotics” and “Combined use of antibiotics” are still a non-negligible problem in antibiotic prescription overuse.

In our study, the majority of inappropriate antibiotic prescriptions were prescribed by physicians older than 40 years, with lower professional titles (resident physician / attending physician) and more than 40 years of work duration. Their education was mostly non-undergraduate, and their professional knowledge and experience are often inadequate. Based on this result, it may be necessary to provide refresher courses in antibiotic prescribing for these primary care physicians [47, 48]. Training should emphasize avoiding incorrect and unnecessary use of antibiotic prescriptions in children.

In this study, children insured by new rural cooperative medical system were more likely to be prescribed inappropriate antibiotics than those who had to pay fully out-of-pocket. One possible reason is that many antibiotics in China are included in the National Essential Medicine List [49]. The children enrolled in the new rural cooperative medical system can use these antibiotics for free or partially free. This increases the risk of inappropriate use of antibiotics. Therefore, it is necessary to educate the physicians and patients about the dangers of inappropriate use of antibiotics, so as to establish a correct concept of medication.

The proportion of antibiotics prescribed inappropriately was higher in the third and fourth quarters compared to the first quarter of the year. This may be due to the higher incidence of infectious diseases in autumn and winter [50]. However, most of treated patients were diagnosed with viral infectious diseases.

We also found that inappropriate antibiotic prescriptions in children may be correlated with sex of children. This may be related with the fact that left-behind children in rural areas of China are often cared for by poorly educated grandparents, as well as sex discrimination. In addition, children aged 0–1 were more likely than any other age group to be prescribed inappropriate antibiotics. In our team’s preliminary survey of primary care institutions in Guizhou Province, we found that the educational level of child caregivers was low and were unaware of the dangers of antibiotic resistance. They generally believe that antibiotics are a panacea. However, infants have an immature immune function, weak anti-infection ability, and are prone to various diseases [51]. In order to heal infants as quickly as possible, the caregivers often ask the physicians for prescriptions of antibiotics [52]. This may increase the risk of inappropriate antibiotic use in the infants. Therefore, more information about antibiotic use, such as easy-to-understand brochures and learning videos, should be provided to caregivers of children.

In China, during the COVID-19 pandemic in 2020, the National Health Commission noted that primary care institutions had the lowest level of access and treatment conditions than secondary and above hospitals. Therefore, they were not eligible for COVID-19 treatment. All suspected febrile patients are transferred to a secondary or higher-level hospital for treatment. Therefore, there were no febrile patients in outpatient clinics of primary care institutions. No COVID-19 related patients were seen in this study.

Our study has several limitations. First, the study subjects in primary care institutions may not fully represent the general population of children in China. Second, the time frame of the survey was limited to 1 year, thus we could not judge whether the prevalence of pediatric diseases and antibiotic use differed over several years [53, 54]. Third, the primary care institutions in Guizhou Province generally do not do laboratory testing; the physicians give drugs by experience. Therefore, we cannot find relevant content for further analysis in HIS system. Fourth, due to being unable to obtain more etiological information, the clinical pharmacists in our team can only assume that “the cause of acute pharyngitis and acute tonsillitis is Group A Hemolytic Streptococcus infection (GABHS)”. Hence, the unnecessary use antibiotics may be underestimated.

## Conclusions

The inappropriate use of antibiotics in children is still prominent in primary care institutions in Guizhou, China. Unnecessary use of antibiotics for many diseases and the inappropriate use of lincosamides and aminoglycosides in children in primary care were the main clinical problems of rural children. The education and training of physicians and caregivers in primary care institutions should be strengthened.

## Supplementary Information

**Additional file 1.** Catalogue for Clinical Application of Antibacterial Drugs.

**Additional file 2.** National Health Commission of China for Guiding Principle of Clinical Use of Antibiotics.

## Data Availability

The datasets used and/or analysed during the current study are available from the corresponding author on reasonable request.

## Abbreviations

ABR: Antibiotic resistance
ICD-10: the 10th Edition of the International Classification of Diseases
CDC: Centers for Disease Control and Prevention
HIS: Hospital Information System
LWTC: LianKe Weixin Co., LTD.
OR: Odds ratio
CI: Confidence interval
Ref: Reference group
