# Supplementary material for: Antibiotic prescribing patterns at children’s outpatient departments of primary care institutions in Southwest China
Source: BMC Prim Care. 2022 Oct 26;23:269. doi: 10.1186/s12875-022-01875-9 (PMC9607730; doi:10.1186/s12875-022-01875-9)
Supplement: Supplementary file 1 — Additional file 1. Catalogue for Clinical Application of Antibacterial Drugs. [file 12875_2022_1875_MOESM1_ESM.pdf]

Catalogue for Clinical Application of Antibacterial Drugs

category	Unrestricted class	Restricted class	Special class
Penicillins	Penicillin	Azlocillin	Pipercillin/ Sulbactam
	Oxacillin	Mezlocillin	Pipercillin/ Tazobactam
	Amoxicillin	Ampicillin/ Sulbactam	Mezlocillin/ Sulbactam
	Ampicillin	Ticarcillin/ Clavulnic acid	Amoxicillin/ Sulbactam
	Piperacillin	Sulbenicillin	Amoxicillin/ Fluloxacillin
	Amoxicillin/ Clavulanic acid	Flucloxacillin	Ampicillin/ Cloxacillin
	Procaine Benzylpenicillin	Ticarcillin	Meloxicillin/ Tazobactam
	Penicillin V		Ticacillin/ Tazobactam
	Cloxacillin		Lenampicillin
	Carbenicillin		Nafcillin
			Sultamicillin
			Furbucillin
			Dicloxacillin
Cephalosporins	Cephalexin	Cefotaxime	Cefpirome
	Cefazolin	Ceftazidime	Cefepime
	Cefuroxime	Cefixime	Cefminox
	Ceftriaxone	Cefdinir	Latamoxef
	Cefadroxil	Cefoperazone/ Sulbactam	Cefoperazone/ Tazobactam
	Cefradine	Cefotiara	Ceftriaxone/ Sulbactam
	Cefaclor	Cefathiamidine	Ceftazidime/ Sulbactam
	Cefprozil	Ceftizoxime	Cefotaxime/ Sulbactam
		Cefoxitin	Cefpiramide
		Cefpodoxime	Cefoselis
		Cefoperazone	Cefapirin
			Cephalothin

category	Unrestricted class	Restricted class	Special class
			Cefamandole
			Cefodizime
			Cefetametester
			Cefonicid
			Cefteram pivoxil
			Cefmenoxime
			Ceftibuten
			Loracarbef
			Cefuroxime/ Sulbactam
			Cefotaxime/ Tazobactam
			Ceftriaxone/ Trizobactam
			Ceftazidime/ Sulbactam
Other β-lactams		Aztreonam	Sulbactam
		Cefmetazole	Flomoxef
			Cefotetan
Carbapenems		Ertapenem	Meropenem
			Imipenem/ Cilastatin
			Faropenem
			Panipenem/ Betamipron
			Biapenem
			Panipenem
			Doripenem
Aminoglycosides	Amikacin	Etimicin	Netilmicin
	Gentamicin	Tobramycin	Isepamicin
	Streptomycin	Spectinomycin	Micronomicin
		Neomycin	Paromomycin
			Sisomicin
			Dibekacin
			Arbekacin
			Ribostamycin
Amide alcohols		Chloramphenico l	

category	Unrestricted class	Restricted class	Special class
Tetracyclines	Tetracycline		Tigecycline
	Minocycline		
	Doxycycline		
	Oxytetracycline		
Macrolides	Erythromycin	Dirithromycin	Telithromycin
	Erythromycin estolate	Clarithromycin	
	Erythromycin ethylsuccinate	Azithromycin	
	Acetylspiramycin		
	Roxithromycin		
	Josamycin		
	Kitasamycin		
	Midecamycin		
Glycopeptides			Vancomycin
			Norvancomycin
			Teicoplanin
Lincosamides	Clindamycin		
	Lincomycin		
Quinolones	Norfloxacin	Gemifloxacin	Fleroxacin
	Ciprofloxacin	Lomefloxacin	Pazufloxacin
	Ofloxacin	Moxifloxacin	Sparfloxacin
	Pipemidic acid	Levofloxacin	Enoxacin
			Pefloxacin
			Rufloxacin
			Gatifloxacin
			Antofloxacin
Nitrofurans	Nitrofurantoin		
	Furazolidone		
Nitroimidazoles	Metronidazole	Ornidazole	S-Ornidazole
	Tinidazole		Secnidazole
Sulfonamides	Sulfamethoxazole and trimethoprim		
	Sulphadiazine		
	Trimethoprim		
	Sulfotrimethoprim		
	Sulfamethoxazole		
Others	Fosfomycin	Colistin	Fusidic acid
		Rifaximin	Polymyxin B
			Linezolid

category	Unrestricted class	Restricted class	Special class
Antifungal drugs	Nystatin	Itraconazole (Oral dosage forms)	Micafungin
	Fluconazole	Ketoconazole	Caspofungin
	Flucytosine		Voriconazole
	Terbinafine		Amphotericin B
	Clotrimazole		Itraconazole (Intravenous formulations)
	Miconazole		Posaconazole
			Anidulafungin
