# Supplementary material for: Antibiotic prescribing patterns at children’s outpatient departments of primary care institutions in Southwest China
Source: BMC Prim Care. 2022 Oct 26;23:269. doi: 10.1186/s12875-022-01875-9 (PMC9607730; doi:10.1186/s12875-022-01875-9)
Supplement: Supplementary file 2 — Additional file 2. National Health Commission of China for Guiding Principle of Clinical Use of Antibiotics. [file 12875_2022_1875_MOESM2_ESM.docx]

**Summary of National Health Commission of China for Guiding Principle of Clinical Use of** **Antibiotics (2015 Edition) related to this study**

1. **The diagnosis of bacterial infection has the indication of the application of antibiotics.**

According to the patient’s signs of symptoms, laboratory tests and imagological examination results to determine the indications for the application of antibiotics.

1. **The cases without pathogenic results can be taken an empirical therapy according to the clinical manifestation.**

For patients diagnosed with bacterial infection clinically, the possible pathogens can be inferred from the infection site, the underlying disease, the incidence, the place of onset, the history of previous antimicrobial drugs and their therapeutic response, and combined with local bacterial resistance monitoring data, before the bacterial culture and drug sensitivity results are known, or when the cultured specimens cannot be obtained. First give the experience of antimicrobial drugs treatment; for patients with negative culture results, further diagnostic measures can be taken according to the effect of empirical treatment and the patient's situation.

1. **The antimicrobial therapeutic regimen was developed by synthesizing of the patients ' condition, pathogenic bacteria and antimicrobial characteristics as following.**

- Choosing, to the extent possible, highly targeted, narrow-spectrum, safe and affordable antimicrobial drugs. Patients with empirical therapy can choose antimicrobial drugs according to possible pathogens and local drug resistance status.
- Generally according to the treatment dose range of various antimicrobial drugs.
- For the majority of patients with mild to moderate infection, oral treatment should be given first, and oral and well-absorbed antimicrobial varieties should be selected without the need for intravenous or intramuscular injections of the drug. Local application of antimicrobial drugs should be avoided as far as possible.
- Antimicrobial treatment varies depending on the infection, it is generally appropriate to use to reach to normal body temperature, symptoms subside 72-96 hours after, there are local lesions need to be used to the infection focus control or complete dissipation.
- Infections that are treatable effectively with a single drug and combination is not required, unless the cases of severe mixed infections, immune defects, drug-resistant bacterial infections that require prolonged treatment, and the use of more toxic antibiotics.

**Appendix: Principles of empirical antibiotic treatment for various bacterial infections**

| **Disease classification** | **Common pathogenic bacteria** | **Main diagnosis and treatment principles** | **Preferred antibiotic** |
| --- | --- | --- | --- |
| Acute bacterial pharyngitis and tonsillitis | Group A hemolytic Streptococcus | The pathogen was diagnosed by pharyngeal swab culture and rapid antigen test (RADT) before administration, and antimicrobial drugs were selected for hemolytic streptococcus infection. | Intramuscular injection of penicillin G, oral amoxicillin |
| Acute tracheal-bronchitis | Most are viral infections, and a few are mycoplasma, chlamydia or pertussis Bordetella infections. | Symptomatic treatment is the mainstay and antibiotics should not be routinely used. | If a few are mycoplasma, chlamydia or pertussis Bordetella infection, macrolides are used. |
| Community-acquired pneumonia | Streptococcus pneumoniae, Mycoplasma pneumoniae, Haemophilus influenzae, chlamydia pneumoniae, etc | Patients with mild symptoms and normal gastrointestinal function can choose oral drugs with good bioavailability. In severe cases, intravenous administration was used, and oral administration was used when clinical symptoms improved significantly and oral administration was possible. | Penicillin, Amoxicillin, Doxycycline, Minocycline, First or second generation cephalosporins, Respiratory quinolones* |
| Periodontitis, pericoronitis, around the apex | Streptococcus mutans | Local treatment is the main, antimicrobial treatment as a supplement. | Oral amoxicillin |
| Viral diarrhea | Rotavirus, Norwalk-like virus, enteroadenovirus, etc | Symptomatic treatment |  |
| Furuncle and carbuncle | Staphylococcus aureus | 1. Mild skin and soft tissue infections generally do not require systemic application of antibiotics, only local use. Topical administration is dominated by antiseptic preservatives (e.g. iodophor), and in rare cases, some antibiotics mainly for topical use may also be used.  2. Moderate, severe or complex skin and soft tissue infections require systemic application of antibiotics. | 1. Mild condition: local treatment mainly, mupirostar ointment, yushi ester ointment  2. Severe condition with sepsis: resistance to the enzyme penicillin such as benzacillin or cefazolin or cefuroxime. Glycopeptides can be selected for MRSA |
| Impetigo | Staphylococcus aureus, group A hemolytic streptococcus | 1. Mild skin and soft tissue infections generally do not require systemic application of antibiotics, only local use. Topical administration is dominated by antiseptic preservatives (e.g. iodophor), and in rare cases, some antibiotics mainly for topical use may also be used.  2. Moderate, severe or complex skin and soft tissue infections require systemic application of antibiotics. | Mupiroxacin ointment for topical use, penicillin, enzyme-resistant penicillin (e.g., benzacillin) |
| Acute cellulitis | A Group of hemolytic streptococcus | It is a systemic, severe or complex skin and soft tissue infection that requires the whole body to be used as an antibiotics. | Penicillin, amoxicillin oral |
| Urinary tract infection (cystitis, pyelonephritis) | Escherichia colii | 1. For patients with initial onset of acute simple lower urinary tract infection, oral medication is preferred, and antibiotics with low toxicity and good oral absorption are appropriate. The course of treatment is usually 3 to 5 days. 2. Patients with acute pyelonephritis accompanied by fever and other obvious systemic symptoms should be given the drug by injection. After the fever, the drug can be given orally. The course of treatment is generally 2 weeks. Patients with recurrent pyelonephritis need a longer course of treatment and special attention should be paid to preventive measures. | 1. Acute pyelonephritis: Ampicillin or amoxicillin or first, second and third generation cephalosporins 2. Recurrent urinary tract infections: Piperacillin/tazobactam or ammonia Benzillin/Sulbactam or Amoxy Linn/clavulanic acid |
| Pelvic inflammatory | Neisseria gonorrhoeae, Enterobacteriaceae, Streptococcus and Bacteroides fragilis, Streptococcus digesticus, Bacillus perfringens and other anaerobic bacteria, as well as Chlamydia trachomatis, Ureaplasma urealyticum and viruses. | The dosage of antibiotics should be enough, the course of treatment should be 14 days, so as to avoid repeated attacks or chronic disease. The initial treatment of severe symptoms should be intravenous administration, which can be changed to oral administration after improvement. | Second or third generation cephalosporins + metronidazole / tinidazole + doxycycline / azithromycin |
| Acute bacterial otitis media | Streptococcus pneumoniae, Haemophilus influenzae and Moraxella catarrh were the most common pathogens | 1. antibiotic treatment should cover Streptococcus pneumoniae, Haemophilus influenzae and Moraxella catarrh, etc.  2. The course of treatment is 7 ~ 10 days to reduce recurrence. | Oral amoxicillin |
| Pathogenic escherichia coli enteritis | 1. Enterotoxin, enterogenicity, enteroinvasiveness 2. Enterobacteriaceae 3. Enterohaemorrhagic | Replenish fluids and electrolytes in time according to clinical conditions. Feces were collected for routine fecal culture, bacterial culture and drug sensitivity test. Combined with clinical conditions, antibiotics were given. If the clinical efficacy is not satisfied, the drug can be adjusted according to the results of drug sensitivity test. Mild cases can be taken orally; The serious case should be intravenous medication, the condition improves and can be oral instead of oral. | 1. Enterotoxin, enterogenicity, enteroinvasiveness: the second and third generation cephalosporins 2. Enterobacteriaceae: the effect of antimicrobial therapy is uncertain 3. Enterohaemorrhagic: not use antibiotics |
| Antibiotic-associated diarrhea or pseudomembranous enteritis | Clostridium difficile | Replenish fluids and electrolytes in time according to clinical conditions. Feces were collected for routine fecal culture, bacterial culture and drug sensitivity test. Combined with clinical conditions, antibiotics were given. If the clinical efficacy is not satisfied, the drug can be adjusted according to the results of drug sensitivity test. Mild cases can be taken orally; The serious case should be intravenous medication, the condition improves and can be oral instead of oral. | Metronidazole |
| Bacterial keratitis | Common pathogens are pseudomonas aeruginosa, Staphylococcus aureus, streptococcus pneumoniae, enterobacteriaceae bacteria. | 1. As soon as clinical diagnosis is made, empirical treatment with antibiotics should be given immediately, and broad-spectrum strong antibiotics should be the first choice. 2. The main route of administration was local eye drop and subconjunctival injection. Patients with a large amount of hyphema should be administered intravenously at the same time. | Levofloxacin / Ofloxacin / Tobramycin |
